# Heavy Metal Free Ag_2_Se Quantum Dot Inks for Near to Short-Wave Infrared Detection

**DOI:** 10.1021/acsami.5c12011

**Published:** 2025-09-11

**Authors:** Shlok J. Paul, Letian Li, Zheng Li, Thomas Kywe, Ana Vataj, Ayaskanta Sahu

**Affiliations:** Department of Chemical and Biomolecular Engineering, 5894 NYU Tandon School of Engineering, 6 Metrotech Center, Brooklyn, New York 11201, United States

**Keywords:** colloidal quantum dots, Ag_2_Se, infrared detector, photodetector, sensing, sustainable

## Abstract

Infrared (IR) photodetection underpins critical applications, from environmental monitoring to national defense and autonomous navigation. Existing technologies derived from epitaxial HgCdTe and InGaAs are constrained by high cost, complex fabrication, and limited scalability. Colloidal quantum dots (cQD) such as HgTe and PbS have emerged as promising solution-processable alternatives, but their reliance on toxic heavy metals impedes broad adoption under increasingly stringent regulatory frameworks. Here, we report high-performance, heavy-metal-free Ag_2_Se cQD photodetectors utilizing a solution phase ligand exchange (SPLE), which produces reproducible devices through a single-step deposition, eliminating the need for multistep layer-by-layer depositions used in conventional solid-state ligand exchange approaches. The SPLE-based photoconductors exhibit responsivities of ∼150 mA/W under a 0.3 V bias in the 800–1250 nm range. We also demonstrate a self-powered photodiode achieving a specific detectivity of 6.5 × 10^10^ Jones at 1200 nm, with a 3 dB bandwidth of 18 kHz and sub-50 μs response times, rivaling those of other heavy-metal-free cQDs. This work positions Ag_2_Se cQDs as a viable, regulatory compliant alternative for near- to short-wave IR detection and offers a strategy for improving performance in larger Ag_2_Se cQDs active in the midwave IR regime.

## Graphical abstract

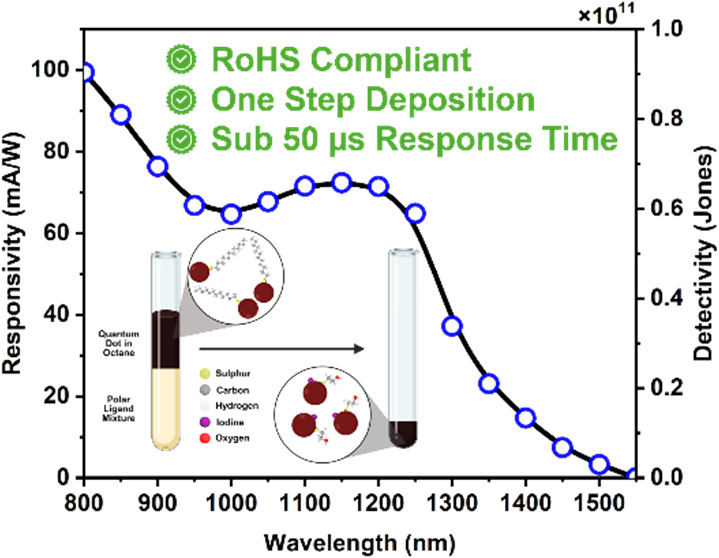

## Introduction

Efforts to extend human vision into the infrared (IR) have propelled key advances in optics and photonics.^1,2^ In parallel, over the past two decades, colloidal quantum dots (cQD) have emerged as promising materials for IR detection, with continued progress now positioning them as candidates for commercial applications.^3–5^ Their versatility enables diverse applications—from greenhouse gas sensing and industrial inspection to autonomous navigation. As the demand for IR detectors grows, so does the imperative to deliver high-performance devices that are cost-effective, easy to manufacture, and fully compliant with governmental regulations.^5–7^

Mercury cadmium telluride (MCT) and indium gallium arsenide (InGaAs) have long served as the foundational materials for infrared detection.^8,9^ However, their reliance on cryogenic cooling, complex processing, and high material costs limits broader deployment.^10^ These challenges open the door for cQDs to enter as a low-cost and scalable alternative.^5–7^ Advances in synthesis and surface chemistry now enable precise control over composition and bandgap, driving cQD devices from early proof of concept detectors to functional IR imaging arrays.^3,5,11,12^

In spite of significant progress, cQD IR detectors—most notably those based on HgTe and PbS—remain constrained by challenges in manufacturing uniformity, environmental stability, and compliance with regulatory standards. In particular, the presence of mercury and lead precludes adherence to Restriction of Hazardous Substances (RoHS) directives. To address the RoHS limitation, a new generation of compliant cQDs—including Ag_2_Te, Ag_2_Se, AgBiS_2_, InSb and InAs—has emerged, appearing to offer performance on par with their heavy metal counterparts in the near-infrared (NIR) and short-wave infrared (SWIR).^13–21^ Among these, silver chalcogenides have garnered increasing interest, particularly in the context of environmentally benign materials for optoelectronic as well as biomedical applications.^22–24^ While Ag_2_Te has been more extensively studied in recent years, Ag_2_Se offers compelling advantages in both sustainability and spectral tunability.^14,20,21,25,26^ The significantly greater earth abundance of selenium compared to tellurium, coupled with the capacity of Ag_2_Se to extend optical response into the midwave infrared (MWIR), positions it as a more scalable, promising and versatile alternative for next-generation IR technologies.^19,27–31^

Surprisingly given the growing interest in environmentally benign IR materials, only a limited number of studies have demonstrated Ag_2_Se-based IR photodetectors operating in the near-infrared (NIR) and short-wave infrared (SWIR) regimes.^32^ Among these, Graddage et al. reported a photodiode exhibiting a responsivity of 4.17 mA/W under reverse bias, although key performance metrics such as detectivity were not disclosed and the overall characterization remained limited.^32,33^ Additionally, Lee et al. employed an aqueous synthesis route to fabricate flexible Ag_2_Se photoconductors, enabling direct formulation into a conductive ink. Their devices achieved responsivities of approximately 3 mA/W under a 5 V bias, with a detectivity of 7 × 10^9^ Jones estimated using the shot noise approximation.^34^

The dearth of work perhaps is because Ag_2_Se cQDs present several challenges during device fabrication. In particular, the conventional thiol based solid state ligand exchanges often leads to undesirable agglomeration and the formation of cracks and voids, all of which contribute to increased photodiode leakage currents.^29,33,35^ In addition, our group has also observed electrochemical instabilities in lateral photoconductors, where metallic silver (Ag^0^) gradually plates onto the ITO electrode with electric fields around 6 V/mm. This observation is consistent with the instability reported by Qu et al.^27^

To address the agglomeration challenges and device fabrication challenges due to the solid-state ligand exchange described earlier, we employ, for the first time with Ag_2_Se, a solution phase ligand exchange (SPLE). This approach allows for the formation of IR active inks that can quickly form crack free films reproducibly and may be deposited using a variety of techniques, including spin coating, spray coating or blade coating, as demonstrated in prior studies with similar inks.^36–38^ In this work, we first demonstrate a simple photoconductor with a responsivity around 150 mA/W under a 0.3 V bias, marking the highest reported performance for an Ag_2_Se-based photoconductor in the 800–1250 nm range. We then develop a self-powered photodiode that achieves a detectivity greater than 6 × 10^10^ Jones from 800–1200 nm. The device also features an 18 kHz 3 dB bandwidth with rise and fall times of around 26 and 40 μs, respectively. These are the highest metrics achieved by Ag_2_Se based photodetectors in the NIR to date.

## Materials and Methods

### Chemicals

The study purchased silver nitrate (AgNO_3_, ≥99.9%), 1-dodecanethiol (DDT, ≥ 98%), selenium pellets (<5 mm, ≥99.99%), trioctylphosphine (TOP, 97%), isopropyl alcohol (IPA, anhydrous, 99.5%), octane (C_8_H_18_, anhydrous, ≥ 99%), hexane (C_6_H_14_, anhydrous, 95%), acetonitrile (CH_3_CN, anhydrous, 99.8%), 2-mercaptoethanol (MpOH, ≥99%), ethanolamine (≥98%), 2-methoxyethanol (anhydrous 99.8%), copper thiocyanate (CuSCN, 99%), diethyl sulfide (DES, 98%) and zinc acetate dihydrate (>98%) from Sigma-Aldrich. Ethanol (CH_3_CH_2_OH, reagent grade), methanol (CH_3_OH, reagent grade), isopropyl alcohol (IPA, reagent grade), acetone (CH_3_COCH_3_, reagent grade), toluene (C_6_H_5_CH_3_, reagent grade) and hexane (C_6_H_14_, reagent grade) were purchased from Greenfield Global. 4-*tert*-butyl-toluene (TBT, 96%) was purchased from Acros Organics. *n*-octane (synthesis grade, >99%) was purchased from Merck KGaA. Deionized water (DI water, 18 MΩ) was fabricated in-house in a Millipore Milli-Q Integral 3 Water Purification System. Silver Iodide (AgI, ≥99.9%) was purchased from Beantown chemicals. All chemicals were used as received without further purification.

### Ag_2_Se Synthesis

NIR-active Ag_2_Se CQDs (∼3 nm in diameter) were synthesized based on an adaptation of routes described in our previous reports.^19,28^ Briefly, 8.75 mL of DDT was mixed with 250 mL of TBT, and 2.565 g of AgNO_3_ was dissolved in 250 mL of DI water in two separate containers. After 30 min of stirring, the solutions were mixed and stirred in the dark for 2 h, forming a milky yellow solution. Upon 20 min settling in a separatory funnel, phase separation was observed with the milky Ag*DDT complex phase on top. The bottom aqueous phase was removed and discarded. The Ag*DDT precursor was transferred to a four-neck flask, connected to a Schlenk line, and purged with nitrogen (N_2_) for 30 min. The solution was then heated to 170 °C, at which point 32.5 mL of 1.0 M TOP:Se diluted with 10 mL of TOP was injected rapidly into the flask. After 60 min growth at 160 °C, the reaction was quenched using a water bath, and 120 mL of ethanol was added to precipitate the cQDs through 5 min centrifugation at 6000 rpm. The precipitate was redispersed in toluene and centrifuged to enable the removal of precipitated impurities. Ethanol was added to the supernatant, and the solution was centrifuged for 5 min at 6000 rpm to precipitate the final product. The cQDs were dried under vacuum and stored in toluene in a dark fridge until further use.

### SPLE Procedure

A stock ligand exchange solution was prepared by dissolving 10 mg of AgI and 1 mL of MpOH in 10 mL of DMF, followed by sonication at 60 °C to promote the dissolution of AgI. The mixture turns a light-yellow color. We centrifuge the solution to remove any unreacted AgI prior to using the ligand mixture.

Separately, 1 mL of Ag_2_Se cQDs, dispersed in toluene, was treated with ethanol, which was added dropwise until turbidity was observed. The mixture was centrifuged at 6000 rpm for 5–10 min, after which the supernatant was discarded, and the resulting precipitate was redispersed in 1 mL of octane.

Subsequently, 1 mL of the octane suspension is combined with 1 mL of the ligand exchange solution and vortexed for 30 s. This process induced phase separation, with the Ag_2_Se cQDs transferring into the lower DMF phase. The upper octane layer was carefully removed and replaced with 1 mL of fresh octane. The biphasic mixture was vortexed again to remove excess nonpolar ligands. This washing procedure was repeated five times. Following purification, a few drops of toluene were added to the DMF phase, and the solution was centrifuged at 6000 rpm for 5 min. The final precipitate was redispersed in DMF to yield a concentrated Ag_2_Se ink with a mass concentration around 300 mg/mL. The ink was then used in device fabrication.

### ZnO Sol–Gel Preparation

500 mg zinc acetate dihydrate is mixed with 142 μL ethanolamine and 5 mL of 2-methoxyethanol overnight in a dark vial in an ambient environment.

### CuSCN Preparation

A 20 mg/mL solution is prepared by dissolving 20 mg of CuSCN in 1 mL of DES. The solution is sonicated for ∼30 min to aid in dissolution and then filtered to remove any undissolved CuSCN.

### Planar Photoconductor Preparation

Interdigitated ITO substrates with 50 μm spacing were obtained from South China Science & Technology Company Limited and underwent a multistep cleaning procedure. First, they were sonicated in a boiling DI water bath containing 1% vol Hellmanex solution for 5 min, followed by additional sonication in neat boiling DI water and then room-temperature DI water for 5 min each. The substrates were then sequentially sonicated in isopropanol and acetone. After drying the substrates with a high pressure dry-air gun they underwent a 30 min ozone cleaning treatment. Neat DMF was dropped on the substrate and spun off at 5000 rpm to prewet the substrate then we add 20 μL of our ink solution and static spin coat at 1000 rpm with 2000 rpm/s acceleration for 5 min. The substrates are then dried under vacuum in a glovebox antechamber before measurement.

### Vertical Photodiode Preparation

Prepatterned FTO substrates were obtained from South China Science & Technology Company Limited and underwent the same multistep cleaning procedure described in the planar photoconductor preparation above. After ozone cleaning the ZnO sol gel was static spuncoat at 3000 rpm for 30s with acceleration of 2000 rpm/s. The substrate was annealed at 200 °C for 10 min after which a second layer was added with the same parameters followed by 30 min annealing at 200C. 20–30 μL of the Ag_2_Se ink is dropped on the substrate and spun at 1400 rpm for 5 min after which it was left to dry under vacuum for 1 h. Then CuSCN is spuncoat at 3000 rpm for 30s and the substrate is dried under vacuum in an antechamber before thermally evaporating 100 nm of silver.

### Photoresponse Measurements

The photoresponse of the sample was characterized using a custom fabricated visible-to-SWIR photoconductivity setup. 300–3800 nm broadband light from an incoherent 250 W Oriel Newport Light source equipped with a halogen bulb was collimated, filtered for second order light and chopped at 25 Hz before being focused onto the input slit of a Cornerstone 260 Vis-NIR extended range 1/4 m monochromator. Based on the selected monochromator wavelength, suitable high pass filters (375, 715, or 1400 nm) were automatically selected. Maximum power output from the monochromator, while maintaining 39 nm resolution, was ensured through adjusting input and output slits to 3 mm. After collimation, the electromagnetic radiation exiting the monochromator was refocused onto the sample mounted in a dark enclosure. An 843-R-USB power meter coupled with a Germanium (Ge) reference detector (818-ST2-IR) was utilized to quantify the wavelength-dependent input power hitting the sample. The generated photocurrent was amplified and converted to a voltage output through a Stanford Research Systems SR570 current preamplifier connected in series with an SR810 lock-in amplifier, allowing for extraction of the photovoltage signal from the light-exposed sample. Sample photovoltage and phase output from the lock-in amplifier were read and saved using a custom-built LabView program.

### Time Response and Bandwidth

To measure the response time of the photodiode, we used the Thorlabs DC2200 LED driver along with a 970 nm M970L4 LED. The sample and LED were placed in an enclosed box a measured distance apart. The incident power was measured using the Newport 843-R-USB power meter. The sample was connected to DLCPA-200 low noise amplifier whose output was sent to a Tektronix TDS 784C oscilloscope. With the DC2200 we can pulse the LED and measure the device output on the oscilloscope. The rise and fall times were obtained by looking at the time taken to reach between 10 and 90% of the response, respectively.

### Setup Calibration with Reference Diode

A calibrated diode the FDS1010 was purchased from Thorlabs. The device was tested identically to our cQD devices using the same photo response and noise measurement setup. The calibrated responsivity and NEP of the devices are available in the diode reference sheet.

### Linear Dynamic Range Measurement

The linear dynamic range was calculated by modulating the LED fluence and using an SR810 lock in amplifier and an SR570 current preamplifier to measure the change in voltage as a function of fluence.

### Noise Measurement

Noise current spectral density is measured using a femto DLCPA-200 preamplifier and a SR770 spectrum analyzer. The device is mounted in a black-out ST500 cryostat with short triaxial cables fed into the preamplifier. 200 Linear averages are used for each scan. All instruments are grounded to a common ground using copper wiring.

### Fourier Transform Infrared Spectroscopy (FTIR)

FTIR spectra are measured in the range of 12000–5000 cm^–1^ and 4000–700 cm^–1^ using a Nicolet iS-50 FTIR spectrometer using the MCT-A/DGTS detector and White/IR sources. Ag_2_Se CQD thin-film samples were prepared by spin coating samples. Clean calcium fluoride or germanium windows were used for background correction.

### X-ray Photoelectron Spectroscopy (XPS)

CQDs were drop-cast on 1 × 1 cm glass substrates and loaded into the XPS vacuum chamber. XPS was used to identify the Ag, Se and I valence states present in the QDs. The photoelectron escape depths are comparable to the size of the QDs studied here. A Versa Probe II XPS system from Physical Electronics with an aluminum (Al) Kα source operated at 50 W and 15 kV with 200 μm spot size was used. Multipak software was used for peak determination.

### X-ray Diffraction (XRD)

Wide-angle XRD was collected from cQDs cast on glass substrates using a Bruker AXS D8 Discover GADDS XRD microdiffractometer equipped with a Co Kα source emitting X-rays at 0.179 nm.

### Atomic Force Microscopy (AFM)

A Bruker Dimension Icon Atomic Force Microscope was used in ScanAsyst Air mode to measure film roughness directly on a spin-cast film in an identical manner to the fabrication of the photoconductors. In this mode, the gain and frequency used during the measurement is optimized by the software.

### Thermogravimetric Analysis (TGA)

Cleaned platinum pans were used in this experiment. The thermal response of the samples in the temperature range 25–500 °C was evaluated using a TGA550 Thermogravimetric Analyzer from TA Instruments with TRIOS software Version 5.4.0.300. Ramp rates were set to 5 °C min^–1^, and a 20 min isothermal hold was performed at 110 °C.

### Transmission Electron Microscopy (TEM)

TEM samples were prepared by filtering dilute concentrations of Ag_2_Se particles in hexane or DMF through a 0.2 μm PTFE/0.22 PES filter and drop casting the filtrate onto lacey carbon-coated copper grids. Images were taken using a Titan Themis 200 S TEM. ImageJ software was used to analyze the particle size.

## Results and Discussion

The solution phase ligand exchange described here follows similar principles to ligand exchanges used for PbS and HgTe cQD systems.^39–42^ Figure 1 is a schematic of the process. The Ag_2_Se cQDs with organic long chain ligands are first dispersed in octane. The dispersion is then transferred to a tube containing DMF and a mixture of MpOH and AgI. A rationale for the selection of ligands is provided in the Supporting Information Section S1. Upon vigorous shaking, rapid ligand exchange occurs with the cQDs transferring into the polar DMF phase. The hydrophobic octane phase is subsequently removed, and the exchanged sample is vortexed with fresh octane multiple times to drive the equilibrium further toward complete exchange as well as to remove any excess native ligands. Toluene is then gently added as an antisolvent, inducing the particles to precipitate upon centrifugation. The precipitated cQDs can subsequently be resuspended in polar solvents (*e.g*., DMF or DFP) at desired concentrations for device fabrication.

**Figure 1:**
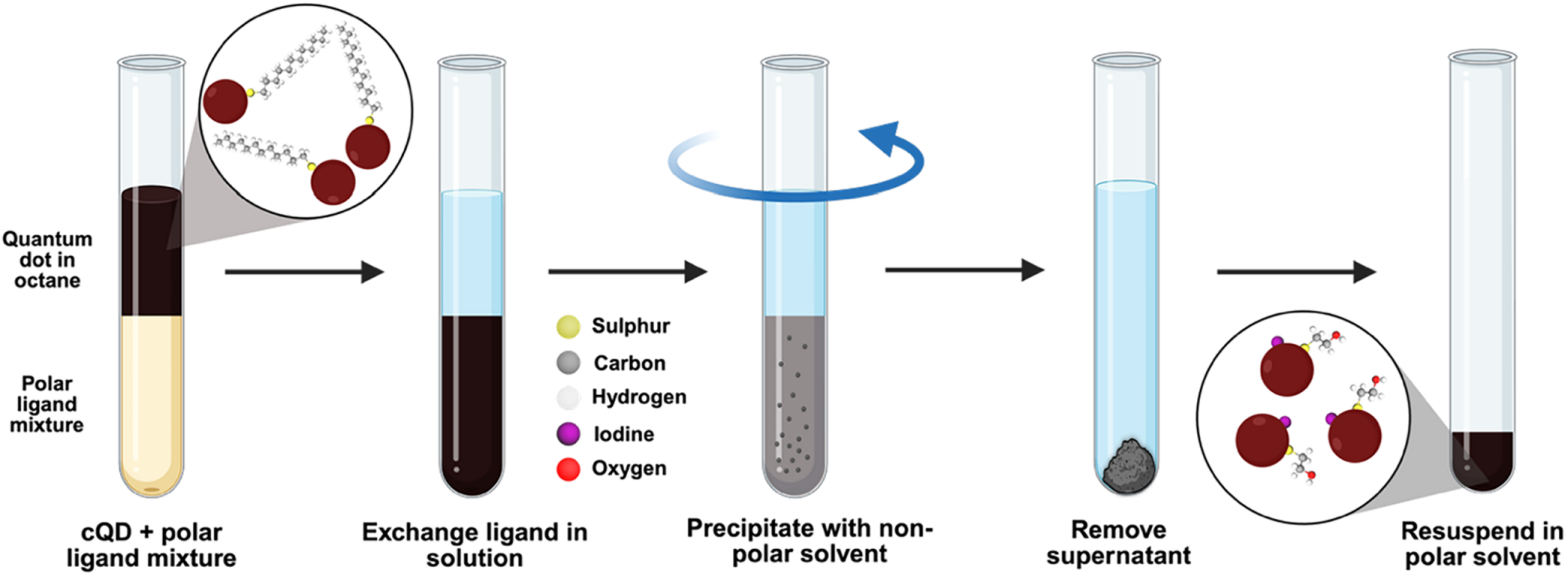
Schematic of the solution phase ligand exchange. The process involves a biphasic ligand exchange followed by precipitation and resuspension steps to end with a photoactive colloidal stable Ag_2_Se ink.

The Ag_2_Se cQDs were synthesized via the reaction of an Ag–DDT complex with TOP–Se, following a method adapted from Mølnås et al.^19^ Representative images of the suspensions during the ligand exchange process are shown in Figure 2a. The absorption spectrum of the as-synthesized (AS) Ag_2_Se cQDs, shown in Figure 2b, exhibits a broad feature spanning 900–1300 nm, which is attributed to inhomogeneous broadening arising from the size distribution.^43^ The impact of the SPLE on the optical absorption is also shown in Figure 2b. A slight red shift of around 30 meV in the optical absorbance is observed following ligand exchange, consistent with prior reports attributing such spectral changes to modifications in surface potential and the dielectric environment surrounding the quantum dots.^44–46^ Evidence for successful ligand exchange is further supported by the IR spectrum shown in Figure 2c where a marked reduction in the C–H stretching modes between 2700 and 3000 cm^–1^ is accompanied by the emergence of a broad O–H band, characteristic of intermolecular hydrogen bonding from MpOH.^47^ To quantify the degree of ligand exchange, we used thermogravimetric analyses (Figure S1). The TGA measurements show that the ligand mass decreases from around 42% in the as-synthesized with long chain surfactants to nearly 13% with the SPLE samples demonstrating effective ligand exchange.

**Figure 2:**
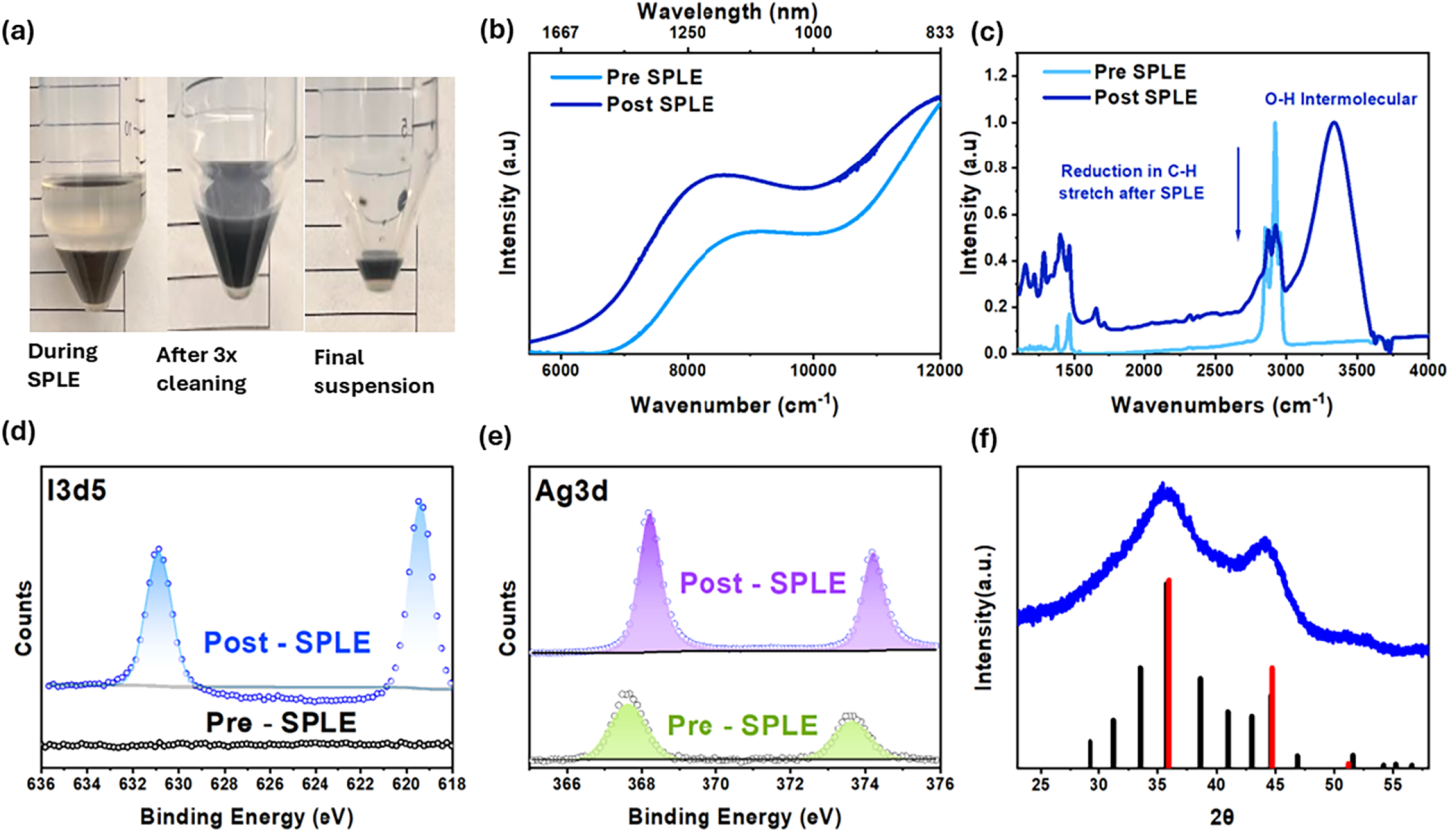
Characterization of Ag_2_Se ink. (a) Images showing the ink through various stages of SPLE, (b) absorption spectrum before and after ligand exchange showing a slight red shift in the first excitonic peak. (c) FTIR spectrum before and after ligand exchange showing a dramatic reduction in C–H stretches and the onset of a O–H peak. (d) XPS spectra showing the development of the I 3d5 peaks after SPLE. (e) XPS spectra showing a slight shift of the Ag 3d peaks, (f) X-ray diffraction spectra of the Ag_2_Se SPLE ink confirming no structural changes after the SPLE. The cubic phase is shown in red (JCPDS: 00-027-0619) while the tetragonal phase is shown in black (JCPDS: 00-006-0501).

To further probe changes in surface chemistry, we use X-ray photoelectron spectroscopy (XPS) as shown in Figure 2d,e. In the Ag_2_Se-SPLE sample the I 3d region reveals new peaks associated with the I^–^ oxidation state, providing evidence for the incorporation of iodide onto the cQD surface. Moreover, the Ag 3d_5/2_ and Ag 3d_3/2_ peaks in the SPLE sample are shifted by approximately 0.5 eV to higher binding energies, consistent with the coordination of surface Ag atoms to more electronegative ligands such as I^–^ and −SH groups.^48^ Structural characterization via X-ray diffraction (XRD), presented in Figure 2f reveals broad peaks typical of Scherrer broadening which limits the definitive assignment of a precise crystal structure post SPLE.^28^ However, the obtained diffraction pattern closely resembles that of previously reported Ag_2_Se QDs synthesized via similar methods indicating that the core crystal structure remains preserved after ligand exchange.^19,28^ This retention of size and morphology is corroborated with transmission electron microscopy (TEM), as shown in Figure S2, where the post-SPLE sample exhibits particle dimensions in agreement with earlier studies via the same synthetic route.^19,28^ More importantly, the SPLE process results in the removal of long chain organic surfactants necessary for charge transport without causing particle aggregation and loss of quantum confinement.

To evaluate the photoconductive performance of our SPLE approach we fabricate lateral photoconductors using interdigitated electrodes with 135 nm of ITO and 50 μm spacings. The samples are spin coated onto the substrate to form a smooth layer of cQDs as shown in the AFM images presented in Figures S3 and S4 with an average thickness of 250–300 nm layer. Figure 3a shows the current–voltage characteristics of the photoconductor which reveal a moderately linear scaling of the photocurrent to the light intensity. If we perform the SSLE instead of SPLE with the same ligand mixture, we see a minor improvement in film conductivity as shown in Figure S5. Time-resolved photoresponse measurements at 970 nm are shown in Figure 3b which exhibit fast switching of around 70 μs rise time and 670 μs fall time under a 0.1 V bias.

**Figure 3:**
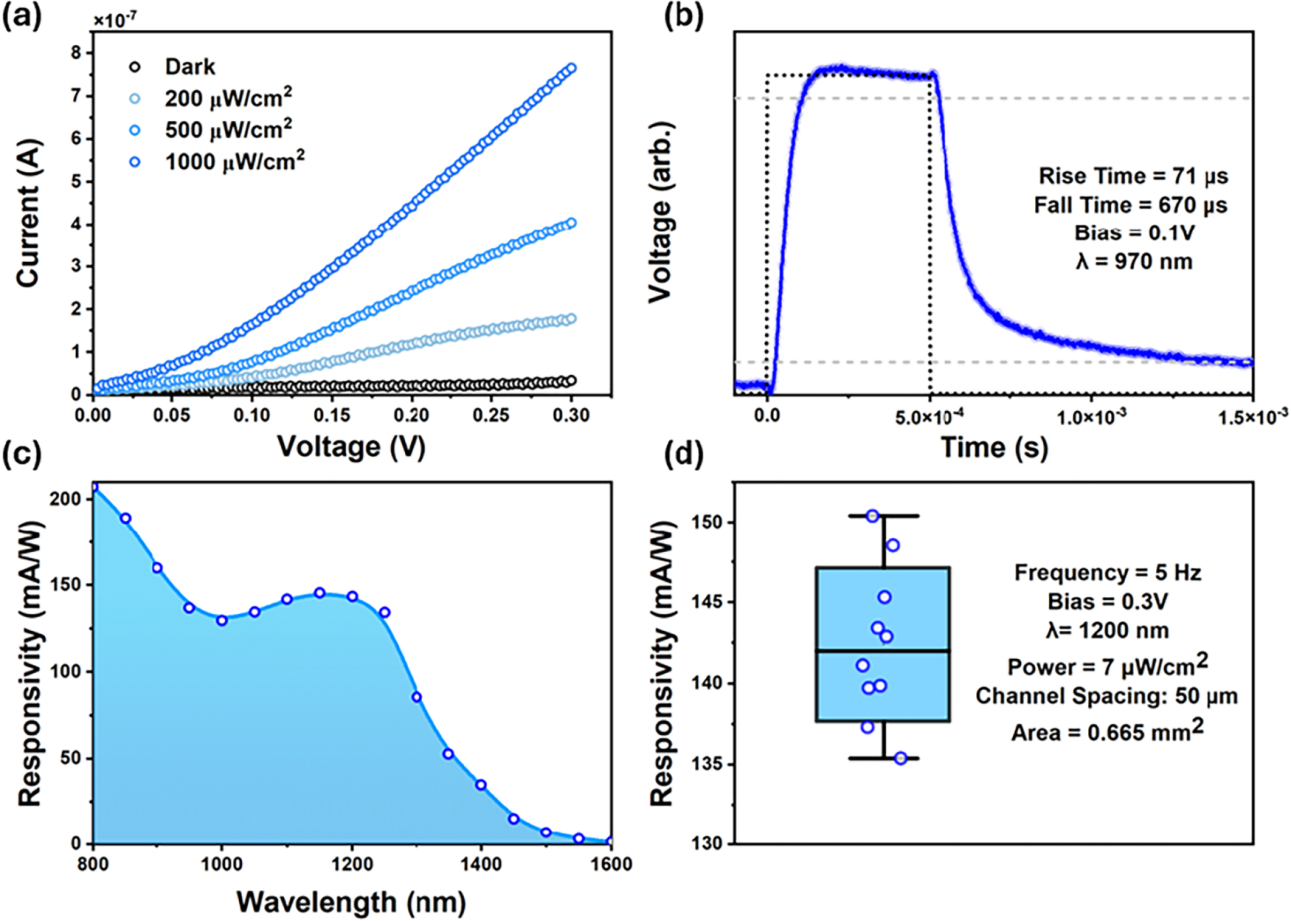
Performance of lateral Ag_2_Se SPLE photoconductor. (a) Current–Voltage curves showing device response to different optical fluences from a 970 nm LED. (b) Device time response at 0.1 V bias showing a ∼70 μs rise and ∼670 μs fall time when pulsed with a 970 nm LED. (c) Spectral responsivity of photodetector at 5 Hz chopping frequency and 0.3 V bias. (d) Responsivity box plot of multiple devices fabricated under the same conditions as in panel (c) to highlight the reproducibility of this method.

The spectral responsivity, measured at 0.3 V and 5 Hz, is shown in Figure 3c. The responsivity is calculated using eq 1 below:^49,50^

1 $$R\left(f,\lambda \right)=\frac{I_{\mathrm{ph}}\left(f,\lambda \right)}{\phi_{\mathrm{in}}\left(\lambda \right)}$$

where *I*_ph_ represents the obtained photocurrent at a specific wavelength and frequency and ϕ_in_ is the incident power at each wavelength. The responsivity qualitatively follows the trend in optical absorption with a responsivity value of around 150 mA/W at 1200 nm corresponding to the first excitonic peak of the cQDs. To our knowledge this is the highest reported responsivity for Ag_2_Se cQDs in the NIR to SWIR region of the electromagnetic spectrum. Comparisons to prior literature are sparse owing to the limited exploration of Ag_2_Se as a photoconductive material and the inherent variability in photoconductor testing conditions across studies.^32^ The most relevant comparison is to earlier work from our group employing a solid-state ligand exchanged Ag_2_Se film with 1,2-ethanedithiol ligands, which exhibited responsivities of ∼25 mA/W at similar biases. However, these devices were prone to poor film uniformity and limited process reliability.^19^ The other comparison is to Lee et al., whose conductive ink Ag_2_Se photoconductors achieve ∼3 mA/W at 5 V bias and limited characterization at 808 nm wavelength.^34^ We believe the high performance stems from the efficient removal of insulating long chain surfactants leading to tight coupling between cQDs in the thin-film device.

To evaluate statistical reproducibility, we measured ten nominally identical devices at 1200 nm shown in Figure 3d, observing a mean responsivity of 142 mA/W with a standard deviation of 4.7 mA/W. While few detector studies report such statistics for colloidal photoconductors, this degree of variation compares favorably with state-of-the-art PbS and HgTe systems, where the reproducibility is obscured by variation in both the active and transport layers.^11,51^

Meaningful comparisons can be made when looking across photodiodes. A table with comparisons across materials is provided in Table S1. Pb and Hg chalcogenide cQDs still set the performance ceiling for solution processed IR detectors in almost every metric. These materials routinely achieve detectivities on the order of 10^12^ Jones, response times below 10 ns, and robust linear dynamic ranges (LDR) exceeding 100 dB, in addition to numerous successful demonstrations of integrated focal plane arrays.^51–54^ Within the RoHS compliant materials Ag_2_Te recently achieved the highest detectivity of a cQD PD around 3 × 10^12^ Jones with 3 μs response times.^13^ InSb and InAs appear to achieve detectivities in the 10^10^–10^11^ Jones with very fast response times from 70 ns to 10 μs and LDR exceeding 100 dB.^16,55–58^

Following successful demonstration of photoconductors, we proceed to fabricate photodiodes based on the SPLE Ag_2_Se cQDs. Our photodiode geometry is shown in Figure 4a where the Ag_2_Se film is deposited in a single step via static spin coating of a concentrated ink. An image of the substrate is provided in Figure S6 showing a dense dark film that spans a 15 mm × 15 mm substrate. The diode current–voltage (I–V) curves under no illumination and illumination are provided in Figure 4b. The shunt and series resistances are approximated by fitting the dark I–V curve to an implicit modified diode equation, eq 2 below, where I_s_ is the dark saturation current, *n* is the ideality factor typically ∼1, *q* is the elementary charge, *k* is the Boltzmann constant, *T* is temperature in kelvin and *R*_s_ and *R*_sh_ are the series and shunt resistances respectively:

2 $$f\left(V,I\right)=I_{0}\left(e^{q\left(V-I\times R_{s}\right)/\mathrm{nkT}}-1\right)+\frac{\left(V-I\times R_{s}\right)}{R_{\mathrm{sh}}}-I$$

**Figure 4:**
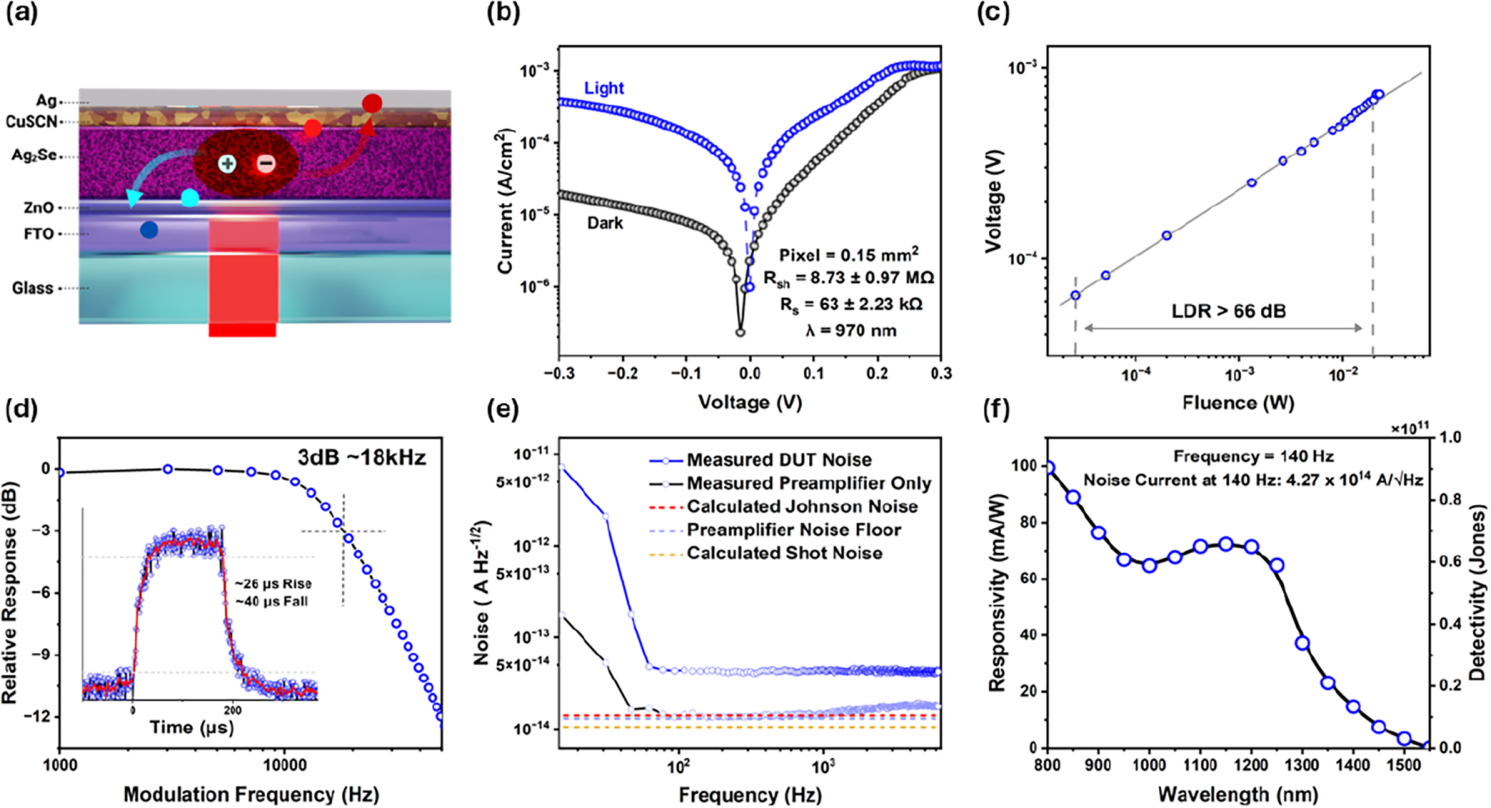
Performance of self-powered Ag_2_Se photodiode. (a) Schematic of diode. (b) Current–voltage sweep for diode with (blue) and without (black) illumination at 970 nm. (c) Linear dynamic range for diode at 970 nm. (d) Frequency response of diode with rise and fall time as inset. (e) Current noise spectral density for sample in dark faraday cage. (f) Responsivity and detectivity vs wavelength for diode measured at 140 Hz.

The series and shunt resistances help assess both the device architecture and interfacial quality. The series resistance reflects the cumulative impedance of carrier transport across the cQD film, heterojunction interfaces and contact resistances. A low series resistance is indicative of efficient charge extraction and minimal voltage drop. The shunt resistance provides a measure of leakage pathways – often arising from film inhomogeneity, pinholes and defects. It is essential to minimize the series resistance while maximizing the shunt resistance to suppress dark current and improve detectivity. From the fit, we obtain shunt resistances that are high ∼8.7 MOhm but also series resistances that are detrimentally high ∼63 kOhm. A recent report from Colbert et al. using PbS and PbS/PbCl_*x*_ core–shell nanocrystals achieves shunt and series resistances around 15 kOhm and 260 Ohms.^59^ Typical InGaAs detectors have shunt resistances ∼10 MOhm at room temperature but benefit from very low series resistances.^60^ The high series resistances can occur from a variety of factors including junction contact resistances, incomplete ligand exchange, poor device fabrication and low carrier mobilities.^61^ Some optimization involving specific choice of chemical species during ligand exchange for efficient surface passivation at the individual cQD level as well as efficient charge extraction with appropriate charge transport layers will be required to improve the series resistances of these Ag_2_Se diodes.

Importantly, the resistive characteristics of the diode influence its linear dynamic range (LDR), a key parameter that governs the fidelity of analog-to-digital conversion and the saturation limit of the diode. A high series resistance limits the linear response of a photodiode under high fluence and can compress its usable dynamic range.^62,63^ The LDR is measured by varying the fluence of the LED and measuring the corresponding device photovoltage. A linear fit is drawn from the lowest power measured (*P*_min_) to the highest power (*P*_max_) that maintains linearity. LDR can be calculated using eq 3 below:^13,64^

3 $$\mathrm{LDR}=10\times \log\frac{P_{\max}}{P_{\min}}$$

Our measurement for LDR, shown in Figure 4c was limited in the lower power ranges by our measurement setup. We obtain an LDR value of ∼ 66 dB which is comparable to values obtained for other heavy-metal free devices.^16,55–57^

In Figure 4d, we report the 3 dB bandwidth as well as the rise and fall times of our diode. These are important parameters since they determine the application speed that the device can be used in. The obtained 18 kHz bandwidth and 26/40 μs rise/fall times are comparable to other heavy metal photodiodes. Even faster devices can be obtained by lowering the device thickness albeit at the cost of sensitivity.^51,65,66^

In order to calculate the detectivity, we measure the noise current spectral density shown in Figure 4e. Our device exhibits relatively low noise currents after 100 Hz roughly around 4 × 10^14^ A/sqrt­(Hz). We include the theoretical shot noise and Johnson noise obtained from our current–voltage curves as well as the preamplifier noise current for this measurement. Figure 4f shows the spectral responsivity and detectivity measured at 0 V and 140 Hz for our device. The detectivity is calculated using eq 4 below:^49,50^

4 $$D^{*}\left(f,\lambda \right)=\frac{R\left(f,\lambda \right)\times \sqrt{A}}{\sqrt{{\left(i_{n}\left(f\right)\right)}^{2}}}=\frac{\sqrt{A}}{\mathrm{NEP}}$$

where *A* represents the pixel area, *i_n_* is the total device noise measured using a spectrum analyzer and the NEP is the noise equivalent power. Lately there have been reports detailing the need for accurate measurement of photodiodes especially with low dimensional materials including cQDs and two-dimensional materials.^67–69^ Being mindful of such reports, we avoid using the shot noise approximation and follow the principle of calibrating our measurement setup against a known photodetector purchased from Thorlabs. We show these calibration measurements in Figure S7 where we achieve measured responsivity values within 5% of the manufacturer specified values. Similarly, our NEP values are off by 0.8 × 10^–13^ $W/\sqrt{\mathrm{Hz}}$ which is reasonably close to the manufacturer reported NEP. These calibration measurements help to confirm the rigor of our measurements and can give the reader a sense of the uncertainties involved in cQD photodiode measurements. We also assessed device stability in terms of dark current and photoresponse measurements. Unencapsulated devices showed fairly decent air stability, maintaining the original dark current values even after 84 h of continuous operation as shown in Figure S8a. We then proceed to test the photoresponse stability by subjecting the photodiode to 1 mW/cm^2^ intensity 970 nm illumination. The device showed no signs of degradation even after 24 h of continuous operation as shown in Figure S8b.

The obtained responsivity of ∼70 mA/W at 1200 nm, 140 Hz and 0 V bias is far superior to previous reports for Ag_2_Se in the NIR ranging around 1–4 mA/W measured at lower chopping frequencies.^19,33^ The calculated EQE which is around 7.4% appears to be on par with unbiased InSb and InAs as shown in Table S1 but far lower than PbS and HgTe. Thus, there exists a potential to improve the EQE of these detectors by employing higher quality cQDs and by modifying charge transport layers.^70^ The detectivity of ∼6.5 × 10^10^ Jones, is to the best of our knowledge the highest reported for Ag_2_Se based cQD diodes in the NIR-SWIR window and within the performance range of InAs and InSb cQDs. With continued advances in the synthesis and surface engineering of Ag_2_Se quantum dots, particularly when coupled with optimized charge transport layers, the SPLE strategy presented here may serve as a foundation for the development of high-performance detectors extending deeper into the SWIR and MWIR spectral regimes.

## Conclusions

In this work, we introduce a simple, environmentally compliant approach to IR photodetection using Ag_2_Se cQDs. By employing a solution phase ligand exchange process, for the first time with Ag_2_Se, we overcome key limitations of solid-state ligand exchange regarding film cracking and reproducibility via facile single-coating step inks. The resultant devices set new performance benchmarks for Ag_2_Se-based systems, with photoconductors achieving reproducible responsivities up to 150 mA/W and photodiodes demonstrating detectivities achieving 6.5 × 10^10^ Jones at 1200 nm with rapid response times, under zero applied bias. These results not only underscore the potential of Ag_2_Se cQDs for next-generation, RoHS-compliant NIR-SWIR devices but also provide a pathway for using such ligand exchanges with MWIR active Ag_2_Se cQDs.

## Supplementary Material



## Notes

TGA analysis of the SPLE process, TEM images of the Ag_2_Se quantum dots, AFM roughness measurements of the SPLE films, Effect of SSLE using ligand mixture, table of summary of other cQD detectors, optical image of fabricated films, calibration detector results and photodiode stability measurements (PDF)
