# Supplementary material for: Heavy Metal Free Ag2Se Quantum Dot Inks for Near to Short-Wave Infrared Detection
Source: ACS Appl Mater Interfaces. 2025 Sep 11;17(38):53735–44. doi: 10.1021/acsami.5c12011 (PMC12464908; doi:10.1021/acsami.5c12011)
Supplement: Supplementary file 1 [file am5c12011_si_001.pdf]

Heavy Metal Free Ag_2Se Quantum Dot Inks for Near to Short-Wave Infrared Detection

Shlok J. Paul[a], Letian Li[a], Zheng Li[a], Thomas Kywe[a], Ana Vataj[a], Ayaskanta Sahu[a,*]

[a] Department of Chemical and Biomolecular Engineering, NYU Tandon School of Engineering, 6 Metrotech Center, Brooklyn, NY, 11201

* Corresponding Author: asahu@nyu.edu (Ayaskanta Sahu)

Supporting Information

Section S1: Motivation to use AgI and MpOH for SPLE

Prior literature with IR HgTe and PbS CQDs showed that the devices with highest photoresponsivity used halide salts in combination with thiol or acetate ligands.[2] The role of the thiol and acetate ligands are not extremely well documented but they are presumed to strip off the native long chain ligands (e.g. oleate) and can act as an intermediate stabilizer during the phase transfer process from the non-polar solvent to the polar solvent.[3] Preliminary experiments involved mixtures of 3-Mercaptopropionic Acid (3-MPA) and Tetrabutylammonium Iodide (TBAI), 3-MPA and Formadinium Ammonium Iodide (FAI), 3-MPA and Methylammonium Iodide (MAI). All of the 3-MPA based solution phase exchanges lead to aggregated dots upon mixing. We reasoned that the -COOH group might be too aggressive during the ligand exchange and can protonate surface metal atoms. For this reason, we switched from 3-MPA to Mercaptoethanol (MpOH) since it was successfully used in the solution phase exchange of HgTe quantum dots. [2] We noticed aggregation when using MpOH with FAI and MAI but obtained stable dots when using TBAI. Unfortunately, devices from these particles demonstrated extremely long response times which we allude to improper removal of excess TBAI and the long chain length and bulky nature of bound TBAI. Then we reasoned that we could use a less bulky iodine source, and the reasonable choice was to use AgI (akin to Pb-halides used for Pb-chalcogenide devices). We hypothesized that Ag ion could passivate any unpassivated surface sites while the I- ion provides colloidal stability. Just using AgI did not result in a ligand exchange and phase change since AgI demonstrated poor solubility in polar solvents like DMF. To assist with the dissolution, we added MpOH as a co-ligand reasoning that the thiolate group in MpOH will help bind with the silver and dissolve the salt. This worked, and we achieved a ligand solution which resulted in very stable inks after SPLE.

Section S2: Thermogravimetric Analysis to evaluate ligand mass change

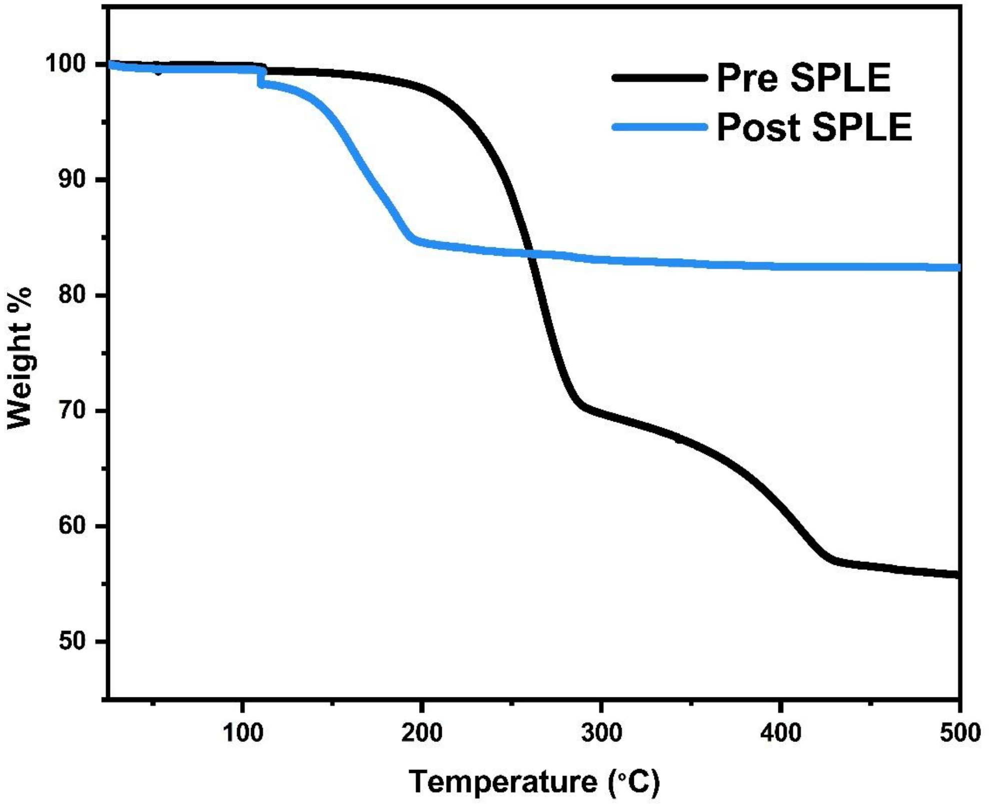

Figure S1. **Thermogravimetric Analysis**. We observed a change in ligand mass between the as-synthesized sample (black) and the SPLE sample (blue) of roughly 42% to 13%.

Section S3: TEM image of sample after SPLE

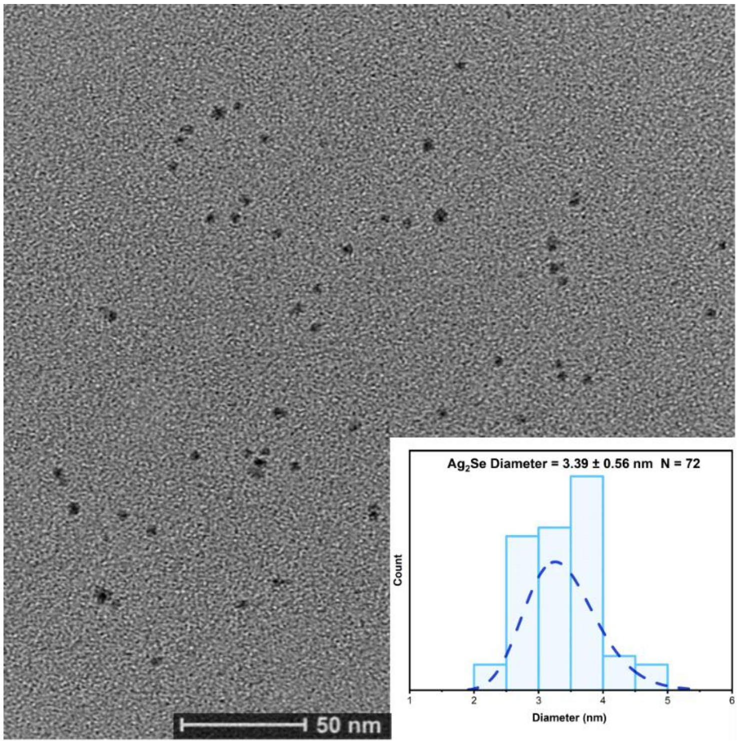

Figure S2. **TEM image of post SPLE sample**. Histogram shows size distribution from multiple TEM images of seventy-two particles in total.

Figure S2 shows a TEM image of the post-SPLE quantum dots drop-cast from a dilute DMF solution. A histogram was generated using ImageJ from multiple images using 72 particles to calculate an average diameter and size distribution. The obtained polydispersity matches that of the synthesis pre-SPLE as shown by Mølnås *et al*. [1] and confirms that the ligand exchange does not result in any drastic changes in size or agglomeration of the quantum dots.

Section S4: Surface roughness of Ag_2Se SPLE film

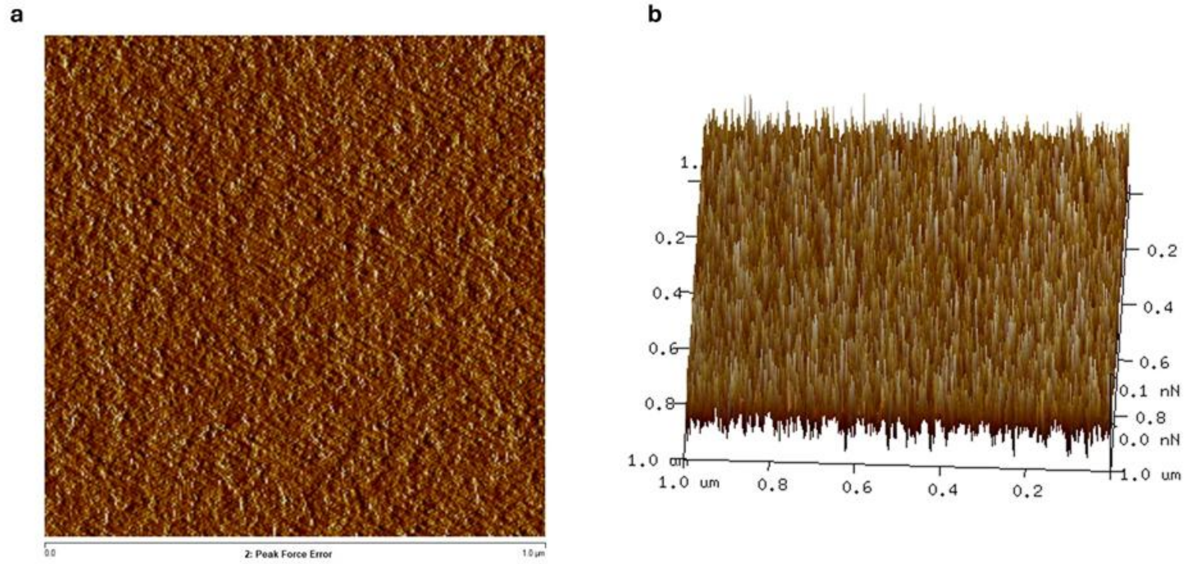

Figure S3. (a) AFM Area scan for 1x1 µm area on Ag_2Se SPLE film. (b) 3-D render of film morphology. The image Ra and Rq values are 1.34 nm and 1.67 nm respectively.

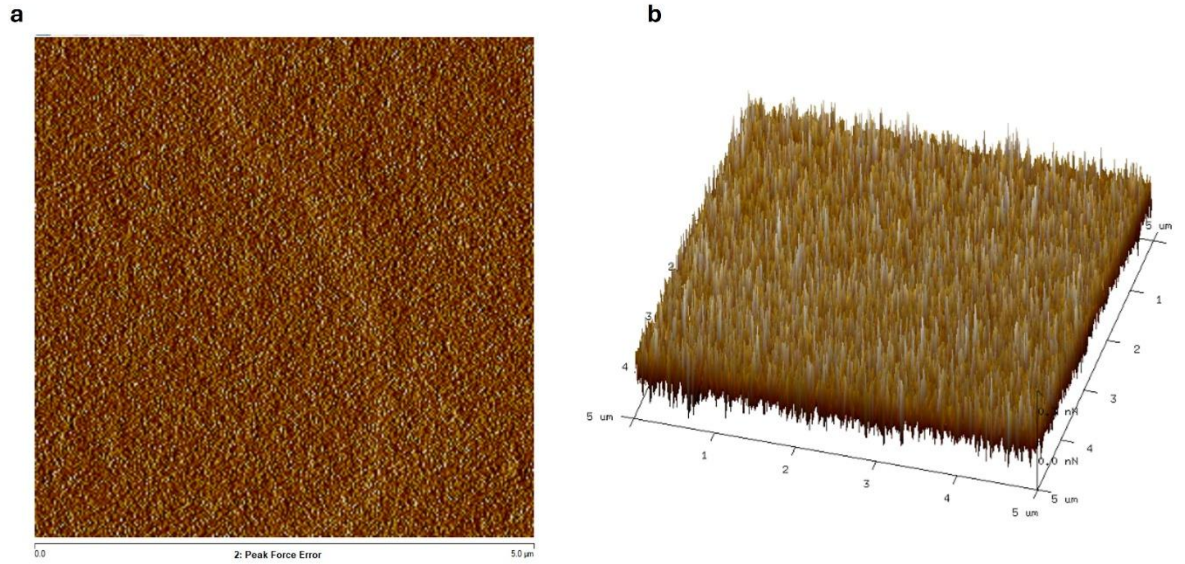

Figure S4. (a) AFM Area scan for 5x5 µm area on Ag_2Se SPLE film. (b) 3-D render of film morphology. The image Ra and Rq values are 2.82 nm and 3.40 nm respectively.

Section S5: Solid state ligand exchange using ligand mixture

To evaluate whether the ligand exchange mixture would work well in the solid-state exchange format we prepared a thin film on 50 um interdigitated electrodes, the same ones used in the main text and performed a solid-state ligand exchange using the same ligand mixture used in the main text. We measure I-V characteristics before ligand exchange and observe extremely insulating films. We then soak the sample in the solution used to perform the SPLE but observe extremely minor improvements to the conductivity of the resulting film.

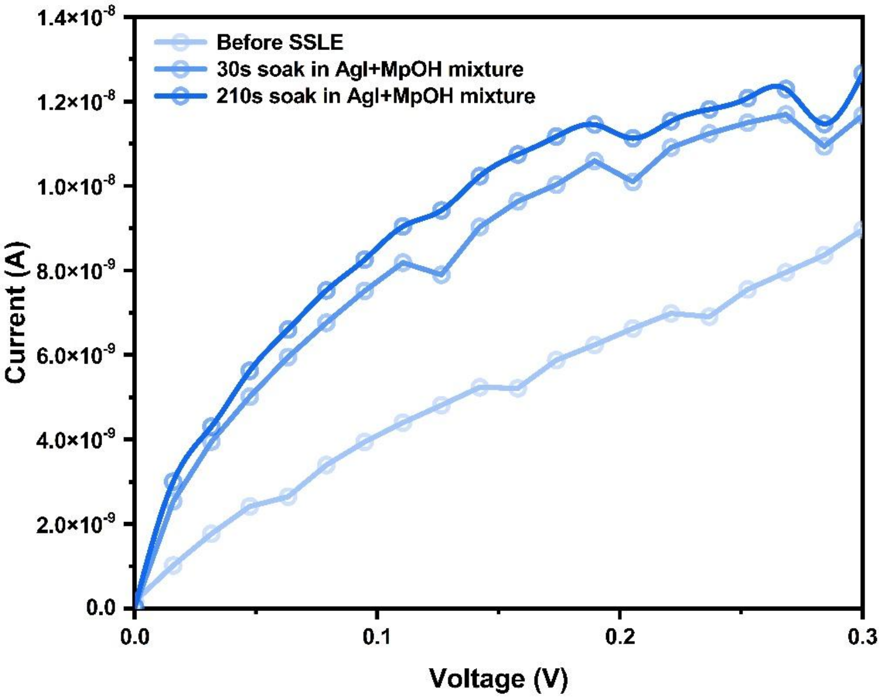

Figure S5: I-V sweep in dark for solid-state ligand exchanged film of Ag_2Se QDs with increasing exchange duration.

Section S6: Comparing our device performance to other reports

Table S1. Summary of device performance of RoHS compliant and non-compliant quantum dot detectors. All device metrics are reported at 298K. PD stands for photodiode while PC stands for photoconductor.

QD Material	Detector Type	Wavelength (nm)	Bias (V)	Rise/Fall Time	D* (Jones)	Responsivity / EQE	Area (mm^2)	Reference
**Ag_2Se**	**PD**	**1200**	**0**	**23/40 µs**	**$6.5x10^{10}$**	**~70 mA/W 7.39%**	**0.15**	**This work**
**Ag_2Se**	**PC**	**1200**	**0.3**	**71/670 µs @0.1V**	**N/A**	**142 mA/W**	**0.665**	**This work**
Ag_2Se	PD	1200	N/A	<240 ms	N/A	4.19 mA/W	49	Graddage et al. 2020[2]
Ag_2Se	PD	1200	0	N/A	N/A	~1 mA/W	6.02	Molnas et al. 2023[1]
Ag_2Se	PC	808	5	~0.5 s	$7x10^9$	~3 mA/W	N/A	Lee et al. 2019[3]
Ag_2Te	PD	2000	0	72 ns	$1x10^7$	0.21%	0.9	Ahn et al 2024[4]
Ag_2Te	PD	1400	0	1.3/3.3 µs	$3x10^{12}$	20%	1	Wang et al 2024[5]
Ag_2Te	PD	1400	0	N/A	N/A	1.5 mA/W 0.14%	49	Ouyang et al 2021 [6]
Ag_2Te	PD	1500	0.3	97/196 µs	$1.4 x10^8$	380 mA/W 30%	1	Yuan et al 2025 [7]
Ag_2Te	PD	1550	0.2	29/50 µs	N/A	27.6 mA/W	2.83	Sharma et al 2025 [8]
InSb	PD	1240	0	70 ns	$4.4x10^{11}$	5%	0.09	Peng et al 2024[9]
InSb	PD	1200	1	8.9 µs	$1x10^{11}$	75%	N/A	Muhammad et al 2023[10]
InAs	PD	1020	1	9 µs	$1.6x10^{10}$	43%	4	Jung et al 2024[11]
InAs/ZnSe	PD	1450	1	1 µs	$1.2x10^{10}$	15%	10	Sheikh et al 2024[12]
PbS	PD	1550	0	~ 10 ns	$8x10^{11}$	80%	0.0078	Vafaie et al. 2021[13]
PbS	PD	1300	2	5 µs	$1.01x10^{12}$	0.38 mA/W 76.6%	0.81	Liu et al. 2023[14]
HgTe	PD	1700	0	~ 8 µs	$3.6x10^{11}$	720 mA/W 56%	N/A	Yu et al. 2024[15]

Table S1 compiles various infrared cQD detectors so that comparisons to the fabricated devices in the paper can be made.

Section S7: Optical image of SPLE film

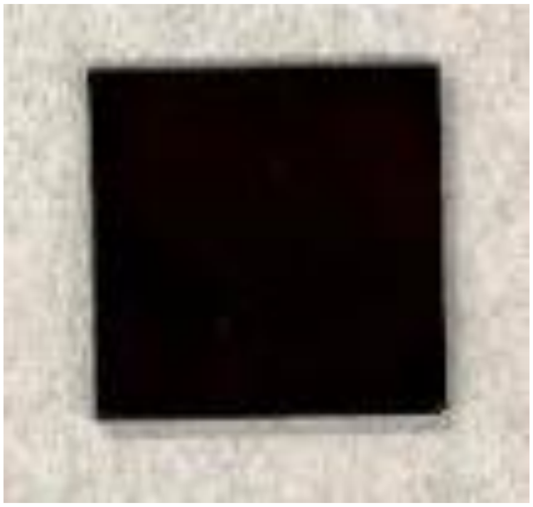

Figure S6. Optical image of film of SPLE Ag_2Se cQDs on ZnO sol-gel layer. The picture shows uniformity of the deposited film.

Figure S6 shows an image of the diode during fabrication. After the ZnO layer is deposited, we deposit the Ag_2Se ink using a static spincoating process. The film produced is an optically uniform dark-colored film similar to those produced using PbS inks.

Section S8: Calibration of photoresponse and noise measurement setup

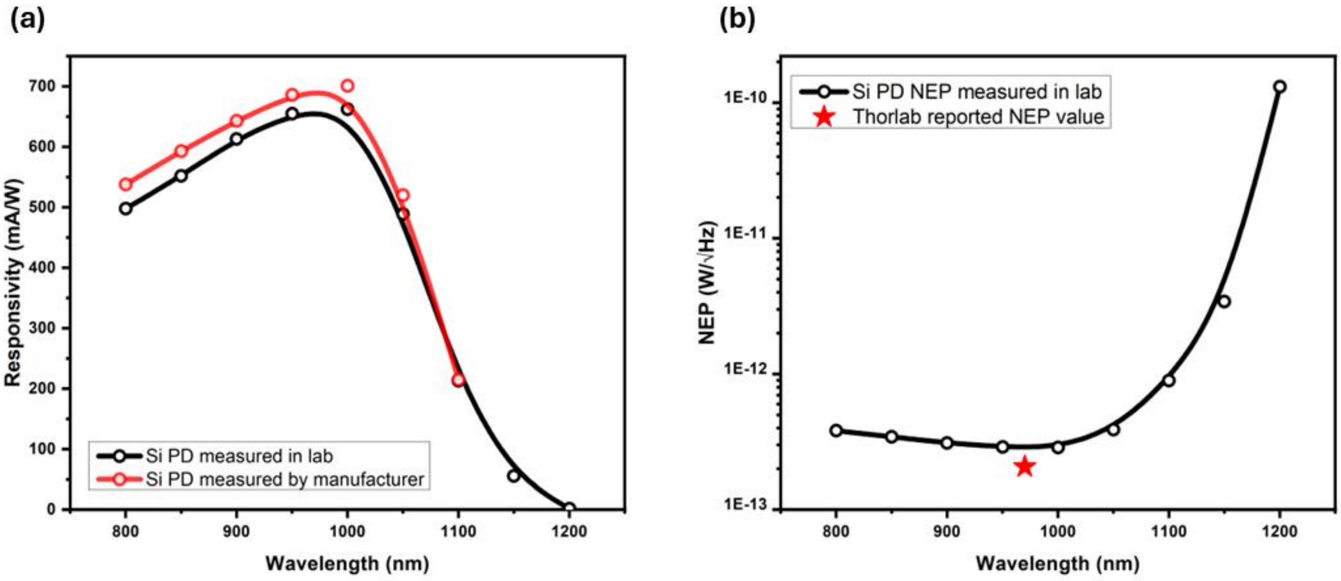

Figure S7. Results for calibration of photoresponse measurements (a) Responsivity and (b) NEP for calibrated Si Photodiode

In Figure S7, we show optical characterization using a calibrated photodiode, the FDS1010 from Thorlabs, to gauge the accuracy of our measurements. The responsivities, shown in Figure S3(a) are off by around 4%-5% in the sub 1000 nm range and can be attributed to inhomogeneities in our beam and slight power measurement errors. We then measure the noise spectral current density of the diode to obtain the NEP spectrum in Figure S3(b) where we are off by 0.8×10^{-13} $W/\sqrt{Hz}$ likely due to the error in the responsivities.

Section S9: Photodiode stability measurements

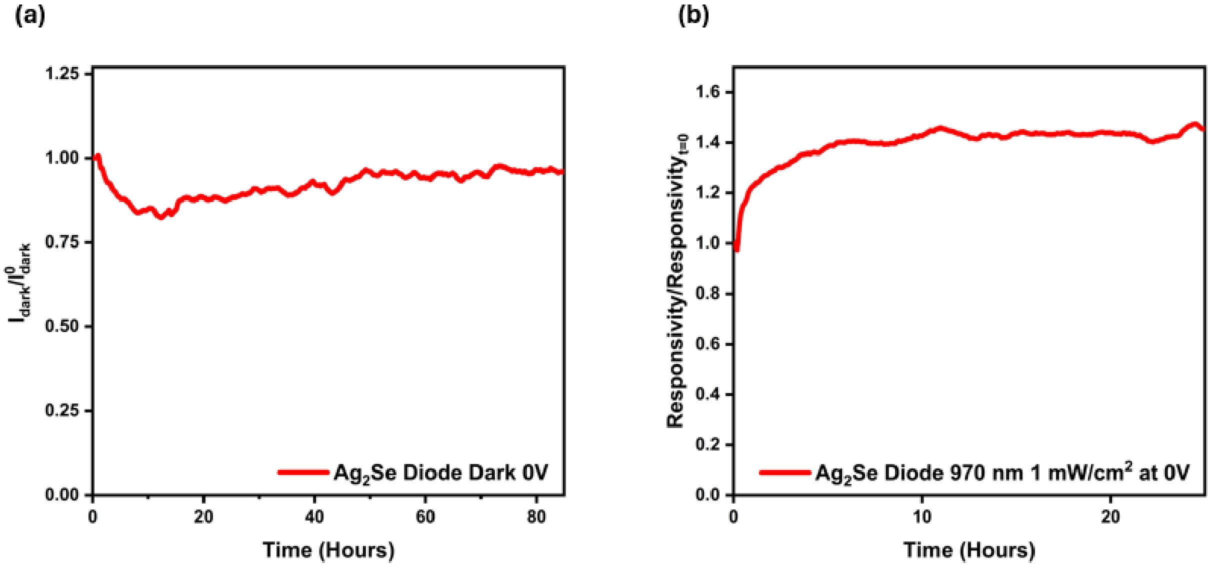

Figure S8: Stability Measurements. Relative change in (a) dark current and (b) responsivity under 970 nm 1mW/cm^2 illumination at 0V applied bias as a function of time in Ag_2Se photodiode.

References

[1] Understanding the growth mechanisms of ultrasmall silver selenide quantum dots for short-wave infrared detectors, Colloids Surf. Physicochem. Eng. Asp. 674 (2023) 131946. https://doi.org/10.1016/j.colsurfa.2023.131946.

[2] N. Graddage, J. Ouyang, J. Lu, T.-Y. Chu, Y. Zhang, Z. Li, X. Wu, P.R.L. Malenfant, Y. Tao, Near-Infrared-II Photodetectors Based on Silver Selenide Quantum Dots on Mesoporous TiO2 Scaffolds, ACS Appl. Nano Mater. 3 (2020) 12209–12217. https://doi.org/10.1021/acsanm.0c02686.

[3] W.-Y. Lee, S. Ha, H. Lee, J.-H. Bae, B. Jang, H.-J. Kwon, Y. Yun, S. Lee, J. Jang, High-Detectivity Flexible Near-Infrared Photodetector Based on Chalcogenide Ag_2Se Nanoparticles, Adv. Opt. Mater. 7 (2019) 1900812. https://doi.org/10.1002/adom.201900812.

[4] Y. Ahn, S.Y. Eom, G. Kim, J.H. Lee, B. Kim, D. Kim, M.-J. Si, M. Yang, Y. Jung, B.S. Kim, Y.J. Chung, K.S. Jeong, S.-W. Baek, Silver Telluride Colloidal Quantum Dot Solid for Fast Extended Shortwave Infrared Photodetector, Adv. Sci. 11 (2024) 2407453. https://doi.org/10.1002/advs.202407453.

[5] Y. Wang, L. Peng, J. Schreier, Y. Bi, A. Black, A. Malla, S. Goossens, G. Konstantatos, Silver Telluride Colloidal Quantum Dot Infrared Photodetectors and Image Sensors, Nat. Photonics 18 (2024) 236–242. https://doi.org/10.1038/s41566-023-01345-3.
[6] J. Ouyang, N. Graddage, J. Lu, Y. Zhong, T.-Y. Chu, Y. Zhang, X. Wu, O. Kodra, Z. Li, Y. Tao, J. Ding, Ag_2Te Colloidal Quantum Dots for Near-Infrared-II Photodetectors, ACS Appl. Nano Mater. 4 (2021) 13587–13601. https://doi.org/10.1021/acsanm.1c03030.
[7] D. Yuan, F. Cao, Z. Han, M. Liu, X. Deng, L. Zhang, X. Liu, S. Cao, X. Liu, Z. Yang, Z. Deng, Y. Li, O. Voznyy, Q. Fan, B. Sun, W. Huang, Zinc Halide Enables Highly Monodisperse Ag_2Te Colloidal Quantum Dots for Short-Wave Infrared Photodetectors, Nano Energy 140 (2025) 111026. https://doi.org/10.1016/j.nanoen.2025.111026.
[8] A. Sharma, D. Yoo, H.-N. Kim, M.-J. Choi, Sequential Surface Tailoring from Colloid to Solid in Ag_2Te Colloidal Quantum Dots Enables High Hole Mobility and Efficient Shortwave Infrared Photodetection, Nano Energy 141 (2025) 111091. https://doi.org/10.1016/j.nanoen.2025.111091.
[9] L. Peng, Y. Wang, Y. Ren, Z. Wang, P. Cao, G. Konstantatos, InSb/InP Core–Shell Colloidal Quantum Dots for Sensitive and Fast Short-Wave Infrared Photodetectors, ACS Nano 18 (2024) 5113–5121. https://doi.org/10.1021/acsnano.3c12007.
[10] Muhammad, D. Choi, D.H. Parmar, B. Rehl, Y. Zhang, O. Atan, G. Kim, P. Xia, J.M. Pina, M. Li, Y. Liu, O. Voznyy, S. Hoogland, E.H. Sargent, Halide-Driven Synthetic Control of InSb Colloidal Quantum Dots Enables Short-Wave Infrared Photodetectors, Adv. Mater. 35 (2023) 2306147. https://doi.org/10.1002/adma.202306147.
[11] B.K. Jung, H. Yoo, B. Seo, H.J. Choi, Y.K. Choi, T.H. Kim, N. Oh, S.Y. Kim, S. Kim, Y. Lee, J.W. Shim, H.Y. Park, G.W. Hwang, T.N. Ng, S.J. Oh, High-Affinity Ligand-Enhanced Passivation of Group III–V Colloidal Quantum Dots for Sensitive Near-Infrared Photodetection, ACS Energy Lett. 9 (2024) 504–512. https://doi.org/10.1021/acsenergylett.3c02515.
[12] T. Sheikh, W.J. Mir, A. Alofi, M. Skoroterski, R. Zhou, S. Nematulloev, M.N. Hedhili, M.B. Hassine, M.S. Khan, K.E. Yorov, B.E. Hasanov, H. Liao, Y. Yang, A. Shamim, M. Abulikemu, O.F. Mohammed, O.M. Bakr, Surface-Reconstructed InAs Colloidal Nanorod Quantum Dots for Efficient Deep-Shortwave Infrared Emission and Photodetection, J. Am. Chem. Soc. 146 (2024) 29094–29103. https://doi.org/10.1021/jacs.4c10755.
[13] M. Vafaie, J.Z. Fan, A. Morteza Najarian, O. Ouellette, L.K. Sagar, K. Bertens, B. Sun, F.P. García de Arquer, E.H. Sargent, Colloidal Quantum Dot Photodetectors with 10-ns Response Time and 80% Quantum Efficiency at 1,550 nm, Matter 4 (2021) 1042–1053. https://doi.org/10.1016/j.matt.2020.12.017.
[14] J. Liu, P. Liu, T. Shi, M. Ke, K. Xiong, Y. Liu, L. Chen, L. Zhang, X. Liang, H. Li, S. Lu, X. Lan, G. Niu, J. Zhang, P. Fei, L. Gao, J. Tang, Flexible and Broadband Colloidal Quantum Dots Photodiode Array for Pixel-Level X-Ray to Near-Infrared Image Fusion, Nat. Commun. 14 (2023) 5352. https://doi.org/10.1038/s41467-023-40620-3.
[15] M. Yu, B. Wang, R. Huang, H. Xia, J. Liu, C. Deng, J. Zhang, L. Gao, J. Tang, X. Lan, Interfacially-Engineered HgTe Colloidal Quantum Dot Photodiodes Using Self-Assembled Monolayer, ACS Photonics (2024). https://doi.org/10.1021/acsphotonics.4c00911.
